# Evaluation of Augmentation Methods in Classifying Autism Spectrum Disorders from fMRI Data with 3D Convolutional Neural Networks

**DOI:** 10.3390/diagnostics13172773

**Published:** 2023-08-27

**Authors:** Johan Jönemo, David Abramian, Anders Eklund

**Affiliations:** 1 Division of Medical Informatics, Department of Biomedical Engineering, Linköping University, 581 83 Linköping, Sweden; 2 Center for Medical Image Science and Visualization (CMIV), Linköping University, 581 83 Linköping, Sweden; 3 Division of Statistics and Machine Learning, Department of Computer and Information Science, Linköping University, 581 83 Linköping, Sweden

**Keywords:** functional MRI, resting state, deep learning, augmentation, autism

## Abstract

Classifying subjects as healthy or diseased using neuroimaging data has gained a lot of attention during the last 10 years, and recently, different deep learning approaches have been used. Despite this fact, there has not been any investigation regarding how 3D augmentation can help to create larger datasets, required to train deep networks with millions of parameters. In this study, deep learning was applied to derivatives from resting state functional MRI data, to investigate how different 3D augmentation techniques affect the test accuracy. Specifically, resting state derivatives from 1112 subjects in ABIDE (Autism Brain Imaging Data Exchange) preprocessed were used to train a 3D convolutional neural network (CNN) to classify each subject according to presence or absence of autism spectrum disorder. The results show that augmentation only provide minor improvements to the test accuracy.

## 1. Introduction

Ever since the emergence of magnetic resonance imaging (MRI) in the 1980s, the absence of ionizing radiation and the flexibility of the acquisition procedure have made this an increasingly important imaging modality in the clinical sciences. The lack of contrast between different tissues in the brain and the interference of the mineralized tissue around it when using X-ray techniques make MRI especially useful in neuroimaging.

While a wide variety of neurological conditions can be diagnosed with MRI, psychiatric anomalies have proven illusive to detect. Presumably, this is because these affect many systems distributed throughout the brain and their manifestations are likely subtle as well as time variant. Furthermore, psychiatric anomalies can vary a lot between subjects. Functional MRI (fMRI) is a technique that seems particularly suited to capture this information, as it generates rich 4D data which can be used for studying brain activity as well as brain connectivity. In this work, it is investigated if deep-learning-based diagnosis of autism from resting state fMRI data can be further improved using 3D augmentation.

### 1.1. Resting State fMRI

Resting state fMRI has since 1995 been used to study brain connectivity [1,2]. A major advantage compared to task fMRI is that subjects can simply rest during the whole experiment, which normally takes 5–10 min (resulting in some 150–600 brain volumes, or put differently some 50,000 time series), instead of performing different tasks such as finger tapping or mental calculations. This makes it possible to include subjects which for some reason cannot perform certain tasks. A simple measure of the connectivity between two locations in the brain, called functional connectivity, is the correlation between the two corresponding time series, but several more advanced methods also exist. To limit the size of the 2D correlation matrix, the correlations are normally calculated between the mean time series of some 100–200 brain parcels (instead of some 50,000 voxels). The brain can be divided according to different (resting state) networks, such as the default mode network and the auditory network, and different diseases often affect specific networks.

### 1.2. Autism

Autism spectrum disorder (ASD) is a disorder characterized by certain features in social communication, and restricted, repetitive, or unusual sensory–motor behaviours [3]. The prevalence of ASD is 1–5% in developed countries [4]. The subject of autism has been studied extensively in recent years, and technology has already contributed to the development of treatments for autism, in terms of rehabilitation and communication.

Due to the lack of reliable biomarkers, the diagnosis is usually based on behaviour, which is very time consuming. Recent work has demonstrated that motor abnormalities can be very informative for detection of ASD [5,6], and that machine learning can be used to shorten the behavioral diagnosis [7]. As ASD results from early altered brain development and neural reorganisation [8,9], it should be possible to derive objective biomarkers from neuroimaging data to aid professionals (paediatricians, psychiatrists, or psychologists) in diagnosising ASD. Here, machine learning can be used to learn informative traits from the high-dimensional fMRI data.

### 1.3. Machine Learning for Diagnosis of ASD

Several large collaborative efforts have been made to collect and share neuroimaging data of healthy controls as well as diseased [10,11]. ABIDE (Autism Brain Imaging Data Exchange) [12] is one such effort that make available data for 539 subjects diagnosed with ASD as well as 573 typical controls. The ABIDE data originate from 17 sites, and the subjects were aged 7–64 years (median 14.7 years across groups). Using machine learning in an endeavour to classify (resting state) fMRI data according to the presence or absence of ASD has become increasingly popular recently. This classification can be performed in several ways, either using estimated functional connectivity network matrices (2D) or using derivatives (3D volumes), such as weighted and binarized degree centrality, as different approaches to compress the 4D fMRI data. In this work, 3D volumes are used, as it is not obvious how to augment network matrices.

The ASD classification problem seems hard in that accuracies seldom rise to more than 70% when the model classifies unseen data [13,14,15,16,17,18]. While 1112 subjects is a very large fMRI dataset, it is still small from a deep learning perspective (for example, the popular ImageNet database [19] contains several million images). To further increase the size of the training dataset, and to make convolutional neural networks (CNNs) robust to transformations such as rotation, data augmentation is often used [20,21]. In previous work. it was demonstrated that 3D augmentation for brain tumor segmentation significantly improves the segmentation accuracy [22]. In this work, the purpose is instead to see if 3D augmentation can help train a better ASD classifier, as well as what kind of augmentation techniques work the best.

### 1.4. Related Work

Several other researchers have used the same ABIDE dataset to train deep learning models for classification [16,17,18,23,24], but do not mention anything about augmentation. In a recent review on deep learning for autism by Khodatars et al. [25], only advanced augmentation techniques, such as generative adversarial methods (GANs), are briefly mentioned, but training a GAN requires a very large dataset to start from and there is very little work published on 3D GANs. Some researchers have employed resampling techniques wherein shorter time series have been cropped out of longer ones [13,14], typically for the double purpose of getting an augmented data set while also eliminating the extra complication of variable length sequences. Ji et al. [26] instead applied augmentation to the estimated network matrices. In our study, by contrast, different preprocessing pipelines are used to extract all relevant information from the time dimension, and manipulate data only in the spatial domain.

## 2. Materials and Methods

### 2.1. Data

Preprocessing of 4D resting state fMRI data is a complex process involving many different steps, and there is no consensus regarding what the optimal pipeline or toolbox is [27]. Head motion is a major problem in resting state fMRI, as it can, for example, result in erroneous group differences if two cohorts differ in the mean amount of head motion [28,29]. All processing pipelines therefore perform head motion correction, and use additional steps to further suppress motion related signal. ABIDE preprocessed [30] (http://preprocessed-connectomes-project.org/abide/, accessed on 10 February 2023) shares preprocessed ABIDE [12] data from structural MRI and resting state fMRI in various forms. As all the preprocessing has been completed, the focus in this work is on the machine-learning-based diagnosis, and other researchers can use the same preprocessed data to reproduce the presented findings. Resting state derivatives (3D volumes where the time dimension has been collapsed into different forms of statistics) resulting from two pipelines were downloaded from ABIDE preprocessed, for 1112 subjects.

One pipeline was the connectome computation system (CCS) [31], which performs slice timing correction, motion realignment, and global intensity normalisation. The data were cleaned from confounders by performing regression with the estimated head movement parameters, the time-dependent global mean intensity, as well as regressors for linear and quadratic drift. Each time series was also band pass filtered (0.01–0.1 Hz). This preprocessing corresponds to the strategy called global_filt. Each subject was, furthermore, registered to the MNI152 brain template using boundary based rigid body registration [32] for functional to anatomical registration, and FLIRT and FNIRT for anatomical to template registration [33].

Another such pipeline was “data processing assistant for resting-state fMRI” (DPARSF) [34]. It also performs slice timing correction and motion reallignment, but does not perform any intensity normalisation. The same confounders are corrected for and the same band pass filtering is performed, whereupon functional to anatomical registration was performed with ordinary rigid body methods and anatomical to MNI152 brain template registration completed using DARTEL [35].

**Figure 1:**
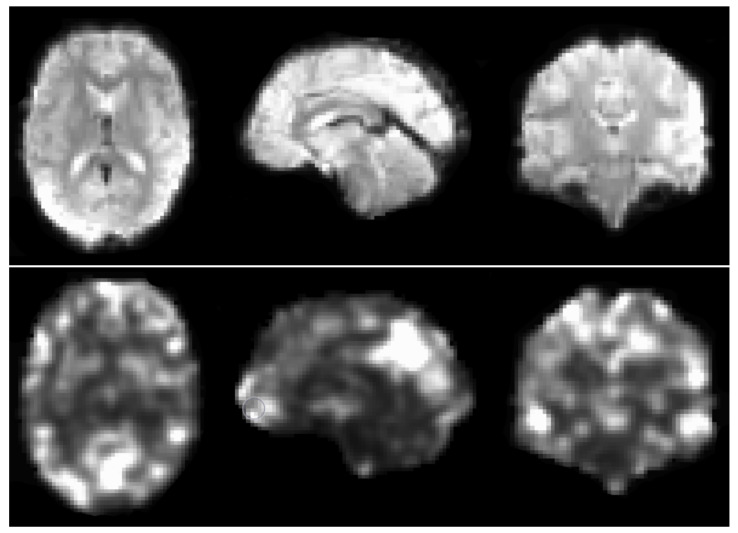
(**Top**): an fMRI volume obtained after preprocessing with the CCS pipeline. (**Bottom**): the REHO derivative obtained from the preprocessed 4D fMRI dataset, used by the 3D CNN to classify each subject as control or ASD. Different types of 3D augmentation were applied to each REHO volume, in an attempt to improve the test accuracy. Several other derivatives are available in ABIDE preprocessed, but were not used in this study due to time-consuming training.

After preliminary testing of the 10 available derivatives available in ABIDE preprocessed (amplitude of low frequency fluctuations (ALFF), weighted and binarized degree centrality, dual regression, weighted and binarized eigenvector centrality, fractional ALFF, local functional connectivity density (LFCD), regional homogeneity (REHO), voxel-mirrored homotopic connectivity (VMHC)), the REHO derivative was chosen for comparing different augmentation strategies. Regional homogeneity is a measure of correlation between a voxel’s time series and those of its neighbours [36], based on the non-parametric rank correlation statistic known as Kendall’s Coefficient of Concordance (KCC) [37]. Each derivative volume from the resting state fMRI data has a size of 61 × 73 × 61 voxels (each 3 × 3 × 3 mm${}^{3}$), which is fed into the 3D CNN described below. See Figure 1 for a preprocessed fMRI volume and the REHO derivative from the CCS pipeline, downloaded from ABIDE (https://s3.amazonaws.com/fcp-indi/data/Projects/ABIDE_Initiative/Outputs/ccs/filt_global/func_preproc/OHSU_0050147_func_preproc.nii.gz, accessed on 1 August 2023; https://s3.amazonaws.com/fcp-indi/data/Projects/ABIDE_Initiative/Outputs/ccs/filt_global/reho/OHSU_0050147_reho.nii.gz, accessed on 1 August 2023). The 539 subjects with ASD and the 573 controls were split 70/15/15 into training, validation, and test sets.

### 2.2. Deep Learning

CNNs are often used for deep-learning-based classification and segmentation of image data, as learning a number of small filters is much more efficient compared to training a dense network (which models the relationship between all pixels in an image, instead of only looking at local correlations). While 2D CNNs are much more common, they are easily extended to 3D as convolution can be performed in any number of dimensions. Unfortunately, existing deep learning frameworks do not support 4D convolutions, which would be required to directly classify 4D fMRI data. The 3D CNN used in this work was implemented using Keras and consists of three convolutional layers (with ReLU activation), max-pooling layers, a dense layer with 16 nodes, and a final one-node layer with sigmoid activation. The first and second convolutional layers contain 8 filters each (size 3 × 3 × 3), and the last convolutional layer uses 16 filters. The total number of trainable parameters in the 3D CNN is approximately 450 k. The CNN was trained with the Adam optimizer with a learning rate of $10^{-5}$ and a batch size of 16. To prevent overfitting, early stopping was used with a patience of 50 epochs. The training was run until validation accuracy did not improve, and the model was then restored to the state when the last improvement was seen. As an alternative, the models were also trained for 150 epochs with no conditional stopping. To obtain more robust estimates of the test accuracy, 10-fold cross validation was used and the mean test accuracy was calculated.

### 2.3. Augmentation

There are many types of augmentation that can be useful in 3D. Rotation, flipping, and scaling (zooming in or out) are common for training 2D CNNs, and can also easily be applied in 3D. Elastic (non-linear) deformations are common when training segmentation networks, but perhaps not as common for classification. Brightness augmentation can for example help if the data have been collected at several different MR scanners, as they normally generate data with different brightness [22].

While 2D augmentation functions are included in many deep learning frameworks such as Keras and Pytorch, the support for 3D augmentation is normally lacking. As mentioned by Chlap et al. [21], many researchers use 2D augmentation even if the data are 3D. The 3D augmentation used here is adapted from that of Cirillo et al. [22] and is written in Python/NumPy [38], without facilities for running on a GPU. The 3D augmentation techniques tested in this study are:

- *Flipping*: flipping of the x-axis or not.
- *Rotation*: rotation applied to each axis with angles randomly chosen from a uniform distribution with range between −7.5 and 7.5 degrees, −15 and 15 degrees, −30 and 30 degrees, or −45 and 45 degrees.
- *Scale*: scaling applied to each axis by a factor randomly chosen from a uniform distribution with range ±10% or ±20%.
- *Brightness*: power-law γ intensity transformation with its parameters gain (*g*) and γ chosen randomly between 0.8 and 1.2 from a uniform distribution. The intensity (*I*) is randomly changed according to the formula: $I_{new}=g\cdot I^{\gamma }$.
- *Elastic deformation*: elastic (non-linear) deformation with square deformation grid with displacements sampled from from a normal distribution with standard deviation σ=2, 4, 6, or 8 voxels [39], where the smoothing is done by a spline filter with order 3 in each dimension.

To investigate the effect of combining different types of augmentation, the CNNs were also trained with the two best-performing augmentation approaches according to the CCS pipeline.

The average training time for a single fold were between five minutes and 2.5 h—depending on the type of on-the-fly augmentation employed, the combination of elastic deformation, and an affine transformation being the slowest–using one Nvidia Tesla V100 graphics card for the early stopping models. For the training with a fixed number of epochs, the average single fold training time was at least 10 min but otherwise in the previously mentioned span. In the longer training runs, it is unlikely that the computation speed was bounded by the speed of the graphics card, as the on-the-fly augmentations were performed on the CPU and could be further optimized. In total, some 600 3D CNNs were trained in order to compare all settings.

## 3. Results

**Figure 2:**
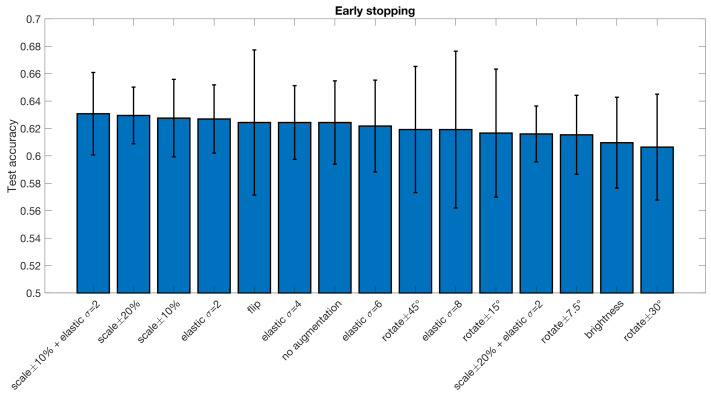
Test accuracy for classifying subjects as healthy or diseased for the ABIDE dataset processed with the CCS pipeline, for different data augmentation approaches. The error bar represents the standard deviation over the 10 cross-validation folds. Note that half of the augmentation approaches result in a test accuracy that is lower compared to the baseline model trained without augmentation, but overall, the differences are small. These results were obtained when using early stopping. Compared to no augmentation, the best augmentation approach increases the test accuracy by 0.6 percentage units.

**Figure 3:**
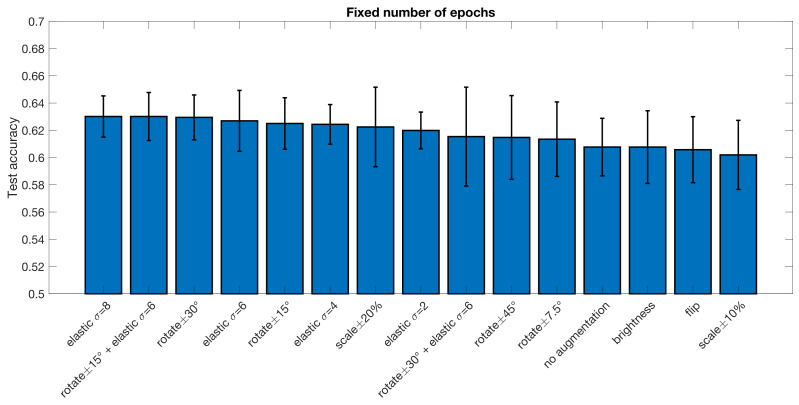
Test accuracy for classifying subjects as healthy or diseased for the ABIDE dataset processed with the CCS pipeline, for different data augmentation approaches. The error bar represents the standard deviation over the 10 cross-validation folds. These results were obtained when using a fixed number of epochs for each training. Compared to no augmentation, the best augmentation approach increases the test accuracy by 2.2 percentage units.

**Figure 4:**
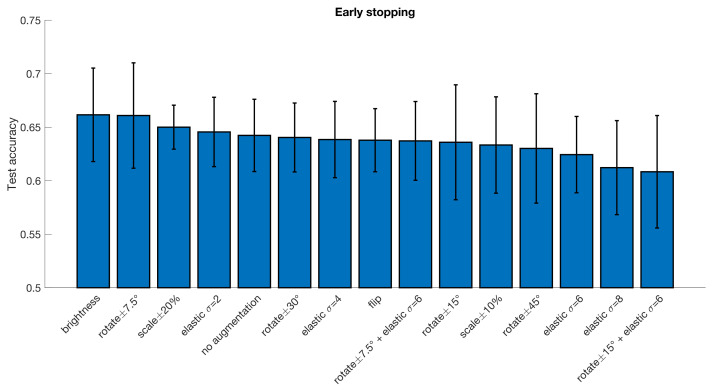
Test accuracy for classifying subjects as healthy or diseased for the ABIDE dataset processed with the DPARSF pipeline, for different data augmentation approaches. The error bar represents the standard deviation over the 10 cross-validation folds. Note that half of the augmentation approaches result in a test accuracy that is lower compared to the baseline model trained without augmentation, but overall, the differences are small. These results were obtained when using early stopping. Compared to no augmentation, the best augmentation approach increases the test accuracy by 1.9 percentage units.

**Figure 5:**
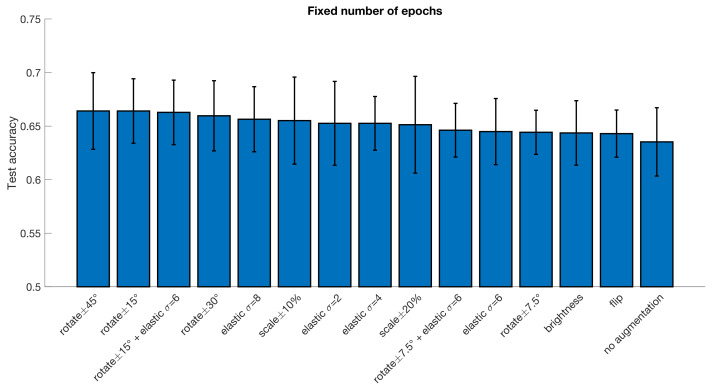
Test accuracy for classifying subjects as healthy or diseased for the ABIDE dataset processed with the DPARSF pipeline, for different data augmentation approaches. The error bar represents the standard deviation over the 10 cross-validation folds. These results were obtained when using a fixed number of epochs for each training. Compared to no augmentation, the best augmentation approach increases the test accuracy by 2.9 percentage units.

The results from all the different augmentation techniques, as well as baseline results obtained without augmentation, are presented in Figure 2 and Figure 3 (CCS pipeline) and Figure 4 and Figure 5 (DPARSF pipeline). As the dataset is balanced (similar number of ASD and control subjects), only classification accuracy is reported (instead of more advanced metrics, such as area under the curve and Matthew’s correlation coefficient). In general, the 3D augmentation does not have a large effect on the test accuracy. For early stopping with the CCS pipeline, random scaling seems to be the best single augmentation approach, but the mean improvement over 10 cross-validation folds is only about 0.5 percentage units. Small elastic deformations also have a small positive effect, while large deformations give worse results.

With the DPARSF pipeline brightness changes appear to be the best augmentation with an increase of 1.9 percentage units, but with high variance over folds, the improvement is negligible. For a fixed number of training epochs, elastic deformations and rotations or combinations thereof seem to work best, with the best improvement of accuracy being 2.2 percentage units in the CCS pipeline and 2.9 percentage units in the DPARSF pipeline. No statistical test was performed to test if this improvement is significant.

## 4. Discussion

Compared to previous work on 3D augmentation for brain tumor segmentation [22], where several 3D augmentation techniques were shown to significantly improve the segmentation accuracy on the test set, only minor improvements of the test accuracy were found in this study (even though the training accuracy is well above 90%, indicating overfitting). Volume classification is in general a problem which requires more training data compared to volume segmentation, as each volume only represents a single training example, which may partly explain the results.

In this study, brightness augmentation only helps for the DPARSF pipeline with early stopping, while it provided a major improvement for brain tumor segmentation for MR images collected at some 20 different sites [22]. A possible explanation is that the data in this study are not raw MR images, since many preprocessing steps have been used to normalize the intensities to a certain range, and to calculate different derivatives. On the contrary, as the ranges of values in the derivative volumes are not, in general, arbitrary in the same way, brightness augmentation can impair the performance. In DPARSF, no intensity normalization is performed, which may explain why the brightness augmentation results are different compared to the CCS pipeline.

Since all the subjects have been registered to MNI space, it was hypothesized that the results may be different if random transformations are applied to the test volumes, but test time augmentation did not change the findings (results not shown). The presented results are for a single preprocessing strategy (global signal regression and bandpass filtering), and a single derivative, and the preprocessing choice can at least in theory affect how much the augmentation helps.

The focus here has been on classifying ASD and controls, with a binary classifier. ASD criteria are based on DSM-5 criteria, and there are currently three levels of severity. It is possible that using 3D augmentation when training a classifier to distinguish the three severity levels could lead to different results.

The conclusion is that 3D augmentation only provides minor improvements in accuracy (0.6–2.9 percentage units) when training 3D CNNs for classification of ASD versus controls, but the results may be different for an easier task where the baseline test accuracy is for example 80%. The results may also differ for other derivatives in ABIDE preprocessed, and when using several derivatives at the same time using a multi-channel 3D CNN. However, to perform the trainings for many combinations of preprocessing, and for different derivatives, would be very time consuming.

## Data Availability

The datasets analyzed for this study can be found at http://preprocessed-connectomes-project.org/abide/, accessed on 10 February 2023.
